# Serological Analysis of Human Pandemic Influenza (H1N1) in Thailand

**DOI:** 10.3329/jhpn.v28i6.6601

**Published:** 2010-12

**Authors:** Slinporn Prachayangprecha, Jarika Makkoch, Sunchai Payungporn, Thaweesak Chieochansin, Chanpim Vuthitanachot, Viboonsuk Vuthitanachot, Apiradee Theamboonlers, Yong Poovorawan

**Affiliations:** ^1^ Center of Excellence in Clinical Virology, Department of Pediatrics; ^2^ Department of Biochemistry, Faculty of Medicine, Chulalongkorn University, Bangkok, Thailand; ^3^ Chumphae Hospital, Chumphae District, Khon Kaen Province, Thailand

**Keywords:** H1N1, Influenza, Serodiagnosis, Surveillance, Thailand

## Abstract

The study was aimed at determining the prevalence of pandemic influenza (H1N1) 2009 among patients with respiratory tract diseases during July-December 2009 using real-time reverse transcription polymerase chain reaction. Haemagglutination inhibition (HI) assay was performed to detect antibody titres against pandemic influenza in 255 medical personnel, 307 members of the general population during the second week of December 2009 in Khon Kaen province, Thailand, and in 100 stored sera collected from people of different age-groups during 2008. The results showed that the pandemic (H1N1) 2009 had occurred during July-December 2009. The results of the HI test after the wave of this outbreak showed that 123 (48%) of the 255 sera collected from the medical personnel, 109 (36%) of the 307 sera obtained from the general population, and only two of the 100 stored sera from 2008 contained antibodies (HI titres ≥40) against pandemic influenza. Antibody against the pandemic (H1N1) 2009 was found in at least one-third of the population. In conclusion, the prevalence of virus and serological data obtained from the study can be used as the serological background level of the Thai population after the July-December pandemic. Finally, the serological data might be useful for outbreak-prevention and control strategies and for the management of vaccination for the pandemic (H1N1) 2009 in Thailand.

## INTRODUCTION

The pandemic influenza (H1N1) 2009 has emerged from North America since mid-March 2009 and rapidly became responsible for the spread of respiratory illness around the world. Since then, it has spread to over 208 countries, and the total number of laboratory-confirmed cases of the pandemic (H1N1) 2009 has exceeded 414,000, with at least 18,114 deaths worldwide (28 May 2010) (1). Research has revealed that the strain of virus is a triple re-assortant among human, avian and swine influenza viruses (2).

Further evidence has also supported that the haemagglutinin protein of this pandemic strain was derived from swine (3). An H1N1 virus was first identified from samples obtained from humans in 1918 when there was an outbreak of pandemic influenza A virus (H1N1) which spread rapidly across the world and caused millions of deaths worldwide. This worldwide outbreak occurred in several waves with increasing virulence (4).

Transmission patterns of influenza are different according to weather conditions, such as temperature and humidity (5). As an example, in the Northern hemisphere, infections mostly occur from November through March whereas infections occur in the Southern hemisphere from July to December, and infections in the tropics tend to be spread throughout the year (6–8). In Thailand, like other tropical countries, the rate of infection due to influenza is normally sporadic throughout the year. It also tends to follow a biphasic seasonal pattern with an increase in the infection rate from June to August and from January to March (9).

The first confirmed cases of pandemic (H1N1) 2009 in Thailand were declared by the Bureau of Emerging Infectious diseases, Department of Disease Control, Ministry of Public Health, on 12 May 2009 (10). The pandemic spread through overcrowded public areas, such as Pattaya and schools in Bangkok, and the virus was probably transmitted from person to person by droplet transmission (11). The frequency of pandemic (H1N1) 2009 infection was generally higher among children and young adults compared to seasonal influenza (12). Antigenic and genetic characterization of the pandemic (H1N1) 2009 virus has shown differences between pandemic (H1N1) 2009 and seasonal H1N1 influenza virus. Hence, the vaccine against human seasonal influenza virus (H1N1) is unlikely to provide cross-reactive immune response against the novel pandemic (H1N1) 2009 virus (13).

To determine the host's immune response, various laboratory methods can be applied, such as haemagglutination inhibition (HI) test, enzyme-linked immunosorbent assay (ELISA), and microneutralization (MN) test. The HI test is a widely-used technique for measuring specific antibody against influenza as it presents a relatively simple and inexpensive method which has been extensively used in epidemiological studies of influenza virus infection.

In this study, we have investigated pandemic H1N1 influenza by performing real-time reverse transcription polymerase chain reaction (rRT-PCR) for the detection of virus in throat or nasopharyngeal swab specimens obtained from randomly-selected patients with respiratory tract diseases. Using the HI test, we have serologically screened medical personnel and the general population in Chumphae district, Khon Kaen province, Thailand, for specific antibodies against the pandemic (H1N1) 2009 virus. Thus, the evaluation of antibody response to the pandemic (H1N1) 2009 among the population might be useful for the management of vaccine. Data will help prioritize certain groups within the population and could also assist in the recommendation for vaccine and management of vaccine strategies.

## MATERIALS AND METHODS

### Sources of specimens

**Fig. 1.**
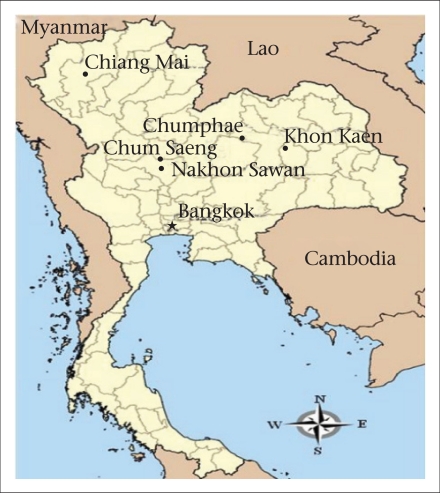
Map of Thailand showing location of Chumphae district

Chumphae in Khon Kaen province was selected as the site for epidemiological studies and surveillance on the pandemic (H1N1) 2009. Chumphae district, with a population of approximately 650,000, was selected for representing a suburban area of Thailand. The district is located in the northeast of Thailand, approximately 449 km from Bangkok (Fig. 1)

Specimens and sera collected from the participants were sent to the Center of Excellence in Clinical Virology, Faculty of Medicine, Chulalongkorn University, within 48 hours. The sera were kept at −20 °C until tested.

### Nasopharyngeal/throat swab specimens

We collected nasopharyngeal or throat swab samples from 20 patients (15 outpatients and 5 inpatients) with symptoms of acute respiratory tract infection, such as fever, sore throat, cough, rhi-norrhoea, nasal congestion. Samples were collected each week during July 2009–December 2009. The specimens were collected in 2 mL of viral transport medium containing antibiotics (penicillin G (2 × 10^6^ U/L) and streptomycin 200 mg/L) kept on ice and sent to the Center of Excellence in Clinical Virology within 48 hours for the diagnosis of influenza infection. All the samples were screened for the pandemic (H1N1) 2009, human seasonal influenza A (H1N1 and H3N2), and influenza B virus by rRT-PCR (14).

### Population for testing of HI antibody

In total, 562 cross-sectional serum specimens were collected from 7 to 15 December 2009 after the July-August wave of the pandemic (H1N1) 2009. People diagnosed with immunodeficiency, bleeding disorder, and any chronic respiratory diseases, such as bronchial asthma, bronchiectasis, or not willing to participate in the study, were excluded. The population to be studied was divided into the following two groups:

#### General population

Three hundred and seven cross-sectional serum specimens were collected from healthy volunteers, devoid of respiratory symptoms, residing in Chumphae district by invitation for screening of antibody against the pandemic (H1N1) 2009. They comprised 185 males and 122 females, with a median age of 30 (range 5–92) years. These serum samples were included in the study protocol to represent the baseline for HI seropositivity in the general population and then used for comparison with healthcare personnel.

#### Healthcare personnel

Two hundred and fifty-five sera were collected by notification from healthcare workers at the Chumphae Hospital, Khon Kaen province, for screening the pandemic (H1N1) 2009. Their median age was 32 (range 19–56) years. Sera were divided into three groups based on their areas of work.

*High-risk group:* Specimens were collected from healthcare personnel who had close contact with patients with respiratory tract disease or collected specimens from patients with clinical respiratory tract infection during the outbreak of the pandemic influenza (H1N1) 2009 or had contact with a person with laboratory-confirmed pandemic (H1N1) 2009.

*Intermediate risk group:* Specimens were collected from healthcare personnel who worked in a high-risk area but were not directly exposed to patients with respiratory tract disease or collected specimens from patients with clinical respiratory tract infection

*Low-risk group:* Specimens were collected from healthcare personnel who work in the hospital but were directly exposed to patients, such as office workers, electricians, secretaries, and accountants.

### Control baseline group

One hundred serum specimens were collected from individuals aged 11–86 years from Chum Saeng district of Nakhon Sawan province (Fig. 1) in March 2008 before the pandemic (H1N1) 2009. Stored serum samples were selected from anonymous specimens with only the age range known. These specimens were collected for a study project on antibody to avian influenza (H5N1).

### Laboratory methods

#### Detection of influenza viruses by real-time RT-PCR

RNA was extracted from 200 μL of each nasopharyngeal swab using the Viral Nucleic Acid Extraction kit (RBC Bioscience Co, Taiwan) according to the protocol of the manufacturer. The extracted RNA was used as a template for the detection of infection due to influenza virus. Primers, specific TaqMan probes, and thermal profiles for the detection were as previously described (12, 14, 15). rRT-PCR was performed using the SuperScript III Platinum One-Step RT-PCR system (Invitrogen, Foster city, CA) in a Rotor-Gene 3000 (Corbett Research, New South Wales, Australia).

#### Propagation of influenza virus

The pandemic (H1N1) 2009 virus used in the study was A/Thailand/CU-H88/09 (accession numbers: HM446345 and HM446344). The influenza virus was propagated by inoculation of virus stock into the allantoic cavity of 10-day old embryonated chicken eggs. The inoculated eggs were placed in an egg incubator at 37 °C for 48 hours. The allantoic fluid was harvested and subjected to centrifugation at 3,000 rpm for 10 minutes. The supernatant was tested for viral titres by haemagglutination assay (HA). Virus-propagation procedures were carried out in a biosafety level 2+ (BSL2+) laboratory.

#### Haemagglutination inhibition assay

To abolish non-specific HIs in serum samples, sera were treated with RDE (receptor destroying enzyme) produced by *Vibrio cholerae* Ogawa type 558 (Denka Seiken, Co. Ltd., Tokyo, Japan) following the specifications of the manufacturer. Briefly, serum and RDE were mixed in a ratio of 1:3 and incubated at 37 °C for 18 hours. Subsequently, the mixture was incubated at 56 °C for 30 minutes to inactivate the RDE and complement system, followed by 10-fold dilution with phosphate-buffered saline (PBS). Two-fold serial dilutions of RDE-treated sera (25 μL) were incubated with eight HA units of pandemic influenza virus (25 μL) per well in a V-shaped 96-well plate (Greiner Bio-One GmbH, Kremsmuenster, Austria) for 30 minutes, followed by addition of 50 μL of 0.5% turkey red blood cells and incubation at room temperature for 30 minutes (16). HI titres of ≥1/40 were considered positive antibody response.

### Statistical analysis

Data were analyzed using the SPSS software for windows (version 17). Chi-square test was used for comparing the different groups.

### Ethics

The Ethics Committee of the Faculty of Medicine, Chulalongkorn University, approved the protocol of the immunological study. Permission for pandemic influenza H1N1 surveillance was granted by the director of Chumphae Hospital, Khon Kaen, to facilitate outbreak control and establish preventive measures. All participants were informed about the objectives of the study, and their written consents were obtained before collection of specimens.

## RESULTS

### Detection of influenza viruses by real-time RT-PCR

**Fig. 2.**
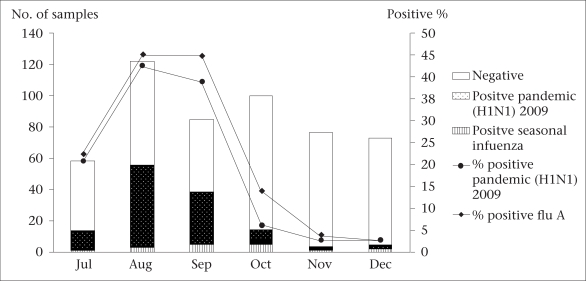
Positive percentage of nasopharyngeal or throat swab samples for pandemic (H1N1) 2009 and human seasonal influenza viruses from Chumphae Hospital, Khon Kaen province, Thailand, between July and December 2009

Five hundred and fourteen specimens were collected from children (233 patients) and adults (281 patients), aged 25 days-86 years. The number of samples and percentage positive for the pandemic (H1N1) 2009 are depicted in Figure 2. From July, the numbers of positive samples increased and reached the highest peak by the end of August 2009 and started to decline from September to December 2009. In contrast, the seasonal influenza strains (H3N2 and influenza B) circulated in a low level throughout the year.

### HI antibodies against pandemic (H1N1) 2009 in healthcare personnel

Based on the HI test performed on 255 healthcare personnel at the Chumphae Hospital, 123 samples (48%) contained specific antibodies (HI titres ≥40) against the pandemic (H1N1) 2009. A comparison between groups of medical personnel showed that the percentage of samples displaying specific antibodies against the pandemic (H1N1) 2009 was 58% (70/120) in the high-risk group, 35% (29/82) in the intermediate risk group, and 45% (24/53) in the low-risk group. Data are summarized in the table. A comparison between antibody-positive risk groups showed a significant (p<0.05) difference between the high-risk group and the intermediate risk group (Table)

**Table.** HI antibody titres against pandemic (H1N1) 2009 among healthcare personnel in Chumphae Hospital, Khon Kaen province, Thailand, 2009

HI titre	High-risk group (n=120) (47%)		Intermediate risk group (n=82) (33%)		Low-risk group (n=53) (21%)	
	No.	%	No.	%	No.	%
20-≤10	50	42	53	65	29	55
40	38	32	15	18	12	23
80	23	19	10	12	9	17
160	8	6	-	-	3	5
320	1	1	3	4	-	-
640	-	-	1	1	-	-

Antibody response between high-risk group and intermediate risk group was significant (p<0.05);

HI titres of ≥40 were used as positive;

HI=Haemagglutination inhibition

### HI antibodies against pandemic (H1N1) 2009 in the general population

**Fig. 3.**
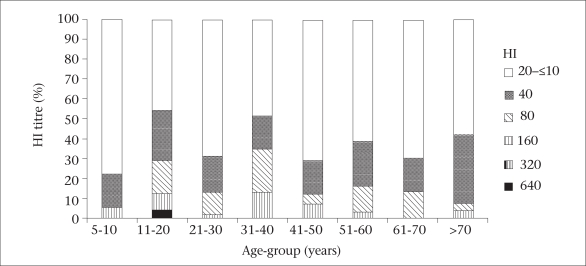
Percentage of HI antibody titres against pandemic (H1N1) 2009 in relation to age among the general population in Chumphae district, Khon Kaen province, Thailand, 2009

The results of the HI test performed on 307 members of the general population, aged 5–92 years (age: 5–10 years: n=18; 11–20 years: n=24; 21–30 years: n=11, 31–40 years: n=23; 41–50 years: n=41, 51–60 years: n=31, 61–70 years: n=30 and over 70 years: n=26) in Chumphae district showed that 109 samples (36%) displayed specific antibodies (HI titres ≥40) against the pandemic (H1N1) 2009. Details of the positive HI antibody response (HI titres >40) among different age-groups are shown in Figure 3.

### HI antibodies against pandemic (H1N1) 2009 in 2008 control group

The results of the HI test showed that two (2%) of 100 anonymous control sera collected during 2008 had HI titres of ≥40 against the pandemic (H1N1) 2009. The positive specimens were derived from two individuals aged over 50 years.

## DISCUSSION

In this study, we have performed the HI assay for determining the antibody levels against the pandemic (H1N1) 2009 to better understand the infection rate and estimated immunity in the population. High numbers of patients diagnosed with the pandemic (H1N1) 2009 were observed during July–September 2009 and then decreased towards the end of the year (Fig. 2). The serum samples collected for HI test in December showed high seropositivity against the human pandemic (H1N1) 2009, implying that approximately one-third of the Chumphae population developed antibodies against the pandemic (H1N1) 2009 at that point in time.

According to a previous study, the HI test in paired serum samples of patients with influenza-like illness (ILI) diagnosed with the pandemic (H1N1) 2009 showed a significant four-fold increase in antibody titres upon comparison between acute and convalescent sera (17). To determine cross-reactivity between seasonal influenza and pandemic (H1N1) 2009, the HI test was performed on acute and convalescent sera of patients with ILI and healthy young adults collected before the outbreak. The results showed that there were no cross-reactivity between antibody against the seasonal and pandemic (H1N1) 2009.

The HI test performed on sera of the medical personnel demonstrated a high percentage (48%) of samples with a positive HI titre against the pandemic (H1N1) 2009. In all probability, these individuals were at a higher risk for infection than the general population due to exposure to clinical specimens of respiratory tract disease and close contact with infected patients.

A comparison between the individual groups of the medical personnel showed that the high-risk group exhibited the highest percentage (58%) of antibody titres whereas the intermediate risk group and the low-risk group presented a lower percentage of antibody titres at 35% and 45% respectively. In the intermediate risk group, the positive HI percentages were lower than those found in the low-risk group. The lower HI titres in the intermediate group might be due to the level of exposure and personal awareness, including knowledge of appropriate protection, such as gloves, respiratory mask, and protective clothing while the low-risk group limited their protection to washing their hands. However, the antibody response in the intermediate risk group did not show a significant difference from that in the low-risk group.

The HI titres among the general population showed that 36% of the Chumphae residents already had developed immune response against the pandemic (H1N1) 2009 virus. Yet, other studies have shown a lower rate of seropositivity (11% and 13% in studies of China and Singapore respectively) (18, 19), which might have been due to the differences in geographic location since various factors, such as overcrowding, climate, and personal hygiene, can affect the transmission efficiency of virus. Hence, the number of people infected with the pandemic (H1N1) 2009 may be different in some areas studied.

The elevated seroprevalence of the pandemic (H1N1) 2009 in the 11–20-year age-group (54%) may be explained by schools serving as gathering places for children and young adults in class-rooms and group activity which create ideal conditions for the transmission of the pandemic (H1N1) 2009 virus, such as lack of or improper handwashing and good personal hygiene (20). The 31–40-year age-group exhibited 52% seroprevalence. These individuals tend to work in air-conditioned offices, use public transport, and attend meetings and other social functions, all of which favour transmission of the virus (21). The elderly have possibly acquired partial immunity since around 1977 when the H1N1 strain with a similar epitope was re-introduced into humans (22). In this study, 42% of the elderly aged over 70 years displayed antibodies against the pandemic (H1N1) 2009, which may have been elicited by previous exposure to a virus containing a similar epitope. Thus, the elderly had acquired some level of immunity against the pandemic influenza A (H1N1) strain. This could explain the lower rate of infection among the elderly people when compared with seasonal influenza and, likewise, why the new pandemic strain tended to affect the younger age-group (12).

In this study, a comparison of the stored serum samples collected in 2008 showed that the HI titre was positive in only two specimens derived from individuals in the age range of 51–60 years and over 70 years. A previous study conducted in the United States established that one-third of people born before 1950 had antibody against the pandemic (H1N1) 2009 (22). However, our baseline data from 2008 revealed a very low prevalence of antibody when compared with the results from sera collected in 2009. This finding was similar to the findings from Japan and China obtained from sera collected before the outbreak (3, 18).

The results of the HI test showed that the population in Chumphae area had developed immunity against the pandemic (H1N1) 2009. This level of immunity among the population can probably lower the infection rate during the next wave due to an effect called ‘herd immunity’ (herd immunity=1-1/R0) (23). Herd immunity can protect non-immunized individuals from infection. The estimated basic reproduction number (R0) of the pandemic (H1N1) 2009 was 1.4–2.1 (24–26). Once the herd immunity reaches 30–50% of the population, it will be sufficient to lower and control the infection rate of the pandemic (H1N1) 2009. As with previous patterns of pandemic, the virus can still cause sporadic infection in non-immunized individuals, although there is no severe outbreak and, at some point, the virus will become a common strain that continually circulates in the human population.

The HI assay was conducted in this study for determining the antibody levels against human pandemic influenza instead of the microneutralization assay. This would provide suitable data on neutralizing antibodies in sera but requires sophisticated laboratories for cell culture. The HI test represents a simpler screening assay and is a less-expensive process, providing acceptable serological data which would be feasible and attractive for large-scale analysis (27).

### Conclusions

Based on the percentage of the pandemic (H1N1) 2009 cases diagnosed and the antibody levels against the pandemic (H1N1) 2009 virus measured, this study has shown that the Chumphae population had gained some level of immunity to the pandemic (H1N1) 2009 during the outbreak between July and December. Thus, the next pandemic (H1N1) 2009 wave may not impact Thailand as severely as suspected, and the disease will become seasonal influenza in the near future. This serological study has provided useful serological data that could help prioritize population groups for vaccination against the pandemic (H1N1) 2009 virus. Moreover, ongoing serological analysis would be essential for the recommendation of vaccine and for the development of strategies to prevent future epidemics or pandemics.

## ACKNOWLEDGEMENTS

The authors express their gratitude to the Commission on Higher Education, Ministry of Education, the Center of Excellence in Clinical Virology, Chulalongkorn University, CU Centenary Academic Development Project, King Chulalongkorn Memorial Hospital, Graduate School of Biomedical Science, Chulalongkorn University, the RGJ PhD Program of the Thailand Research Fund, Ratchadaphiseksomphot Fund, Chulalongkorn University, and MK Restaurant Company Limited for their generous support. This work was also funded in part with federal funds from the NIAID, NIH, Department of Health and Human Services, under Contract No. HHSN266200700007C. They thank Dr. Praveena Kittikhun and Ms Aunyaratana Thontiravong for laboratory assistance. They also thank the staff and nurses of the Chumphae Hospital, Khon Kaen province, Thailand, for their collection of clinical data and specimens. Finally, the authors thank Ms Petra Hirsch for reviewing the earlier version of the manuscript.
